# *Bt* agave: why it is time to explore a new biotechnological frontier

**DOI:** 10.1002/ps.70964

**Published:** 2026-05-23

**Authors:** Aline Vitória Corim Marim, Marcelo Falsarella Carazzolle, Gonçalo Amarante Guimarães Pereira, Carolina Rossi De Oliveira

**Affiliations:** ^1^ Genomics and BioEnergy Laboratory Biology Institute, University of Campinas Campinas Brazil

**Keywords:** *Bt* technology, Cry proteins, genetic transformation, insect resistance, semi‐arid agriculture

## Abstract

Drylands cover 41% of Earth's surface and are expanding due to climate change, requiring innovative agricultural strategies for resource efficiency. Agave, a non‐conventional drought‐tolerant monocot cultivated in Mexico and Brazil, is a sustainable crop with high biomass productivity and low water requirements, supporting industries like biofuels, textiles, and beverages. With high biomass productivity and extremely low water requirements, *Agave* species are increasingly recognized as one of the most sustainable crops for semi‐arid and marginal regions. Despite its environmental resilience, agave cultivation is significantly constrained by insect pests that reduce yield and economic returns. While *Bacillus thuringiensis* (*Bt*) Cry proteins have revolutionized pest management in other crops, their application in *Agave* spp. remains unexplored due to technical barriers in genetic transformation. Here, we argue that the integration of *Bt* technology into *Agave* species is both technically feasible and strategically necessary. We discuss recent advances in plant transformation and regeneration that lower historical barriers in monocots and outline how *Cry*‐based resistance could be incorporated into *Agave* spp. within integrated pest management frameworks. By synthesizing current knowledge and identifying key research priorities, we suggest that *Bt*‐engineered *Agave* spp. could represent a realistic and timely strategy to enhance pest control, sustainability, and resilience in dryland agricultural systems. © 2026 The Author(s). *Pest Management Science* published by John Wiley & Sons Ltd on behalf of Society of Chemical Industry.

## Graphical abstract

Drylands cover 41% of Earth, requiring sustainable crops. *Agave*, drought‐ and heat‐adapted, offers high‐value products with low water needs. Pests limit yield, yet *Bacillus thuringiensis* (*Bt*) Cry proteins, successful in other plants, remain unexploited in *Agave*. Integrating *Bt* offers a feasible strategy for sustainable pest management and resilient dryland agriculture.

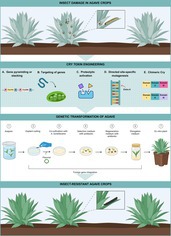

## 1 INTRODUCTION

Species of the genus *Agave*, perennial monocotyledonous plants native to the Americas and classified within the subfamily Agavoideae, are predominantly cultivated in Mexico, Brazil, and other semi‐arid regions.^1^ The genus *Agave* comprises approximately 210 species, among which *Agave tequilana*, *Agave sisalana*, *Agave salmiana*, and *Agave fourcroydes* stand out for their commercial value. These species support diverse industries, including the production of fibers, alcoholic beverages, and bioenergy.^2^, ^3^ Mexico is the world's leading producer of agave‐based beverages, particularly tequila, with an output of 583.5 million liters in 2025.^4^ In 2024, Brazil was the largest global producer of sisal fiber, generating 93 261 tons.^5^ Despite this economic relevance, a significant portion of agave biomass, particularly leaves and processing residues, is discarded, representing untapped potential for energy generation through ethanol and biogas, as well as for the extraction of high‐value compounds.^6^

Adapted to harsh environments, *Agave* species exhibit physiological traits that enable survival in arid climates, with crassulacean acid metabolism (CAM) photosynthesis being central to their exceptional water‐use efficiency and carbon assimilation under drought stress.^7^ Nevertheless, even with such resilience, agave crops remain highly susceptible to insect pests.^8^ The agave weevil (*Scyphophorus acupunctatus*), a coleopteran pest, is the most destructive, frequently causing extensive internal damage and plant mortality, with reported losses reaching up to 90% in heavily infested fields and facilitating secondary pathogen infections.^9^, ^10^ Other pests, including the armored scale (*Acutaspis agavis*) and the maguey red worm (*Comadia redtenbacheri*), also pose substantial risks to agave cultivation.^11^, ^12^ These problems are exacerbated in monoculture systems, where genetic uniformity, limited pest monitoring, and reduced ecological buffering increase vulnerability.^13^

Globally, pest‐related losses affect 20–40% of crop yield reductions in major crops, costing over US$ 70 billion annually.^14^ By 2024, over 30 nations had approved the cultivation of genetically modified (GM) crops, and the global area dedicated to biotech crop production has reached 206.3 million hectares.^15^ Since their introduction in the 1990s, GM crops expressing *Bacillus thuringiensis* (*Bt*) Cry proteins have revolutionized insect pest management, significantly reducing reliance on chemical pesticides and increasing yields in crops such as maize and cotton.^16^ These proteins target specific lepidopteran and coleopteran pests, providing an environmentally friendly alternative to chemical insecticides and becoming a cornerstone of sustainable pest management strategies.^17^

However, the application of *Bt* technology to agave remains unexplored, largely due to its long life cycle, technical limitations in genetic transformation approaches,^2^ and reduced adoption of integrated pest management.^1^, ^18^ These issues are intensified in semi‐arid regions, where historically there are resource constraints,^19^ emphasizing the urgent need for sustainable solutions based on Cry proteins to improve pest control and productivity.

This perspective highlights the promising potential of Cry proteins as a sustainable tool for pest management strategy for agave cultivation in semi‐arid regions. We critically examine the biological and technical barriers that have historically limited genetic transformation in *Agave* spp. and discuss recent advances that may help overcome these constraints. Drawing on preliminary data and lessons from other crops, we outline strategic approaches to develop transgenic varieties with built‐in insect resistance. Such innovations could substantially improve pest control, enhance productivity, and strengthen the sustainability of agave‐based agro‐ecosystems under increasing climate pressure.

## 2 INSECT PESTS IN AGAVE CULTIVATION

During flowering and seed production, agaves plants attract predators and pollinators, expanding the complexity of ecological interactions.^20^, ^21^ In plantations with less intensive management, the presence of other plant species further enhances these interactions, supporting more diverse arthropod communities. In Mexico, at least 273 insect species associated with agaves have been identified, spanning 63 families and seven orders, with a prominence of Coleoptera, Hemiptera, Hymenoptera, and Lepidoptera.^8^ Some of these species are recognized as insect pests (Table 1) or vectors of plant diseases causing severe damage, physiological disorders, or eventually plant death.^8^, ^12^, ^13^

**Table 1:** Summary of the main insect pests associated with agave plants, indicating the species, taxonomic order, and plant parts affected

Species	Order	Part of the plant	Reported symptoms	Reference
*Cyclocephala* spp.	Coleoptera	Root	Stunted growth and root damage	^22^, ^23^
*Macrodactylus* spp.	Coleoptera	Root	No data available	^8^
*Phyllophaga* spp.	Coleoptera	Root	Stunted growth and root damage	^22^, ^23^
*Diabrotica* spp.	Coleoptera	Root	Plant decline with blue‐to‐reddish‐purple discoloration	^23^
*Acentrocneme hesperiaris*	Lepidoptera	Piña or stem	Stunted plant growth, early wilting, necrotic leaf areas, and plant death	^11^, ^23^, ^24^
*Comadia redtenbacheri*	Lepidoptera	Piña or stem	Root damage, reduced vigor, leaf yellowing, and plant death	^11^, ^25^
*Scyphophorus acupunctatus*	Coleoptera	Piña or stem	Vascular damage, plant weakening, reduced piña quality, and plant death^*^	^9^, ^24^
*Dactylopius* spp.	Hemiptera	Piña or stem	Weakening of host plant	^23^
*Strategus aloeus*	Coleoptera	Piña or stem	Tunneling in young plant piñas, growth reduction and plant death^†^	^23^, ^26^
*Acanthoderes funerarius*	Coleoptera	Piña or stem	Root destruction and plant wilting	^23^
*Aonidiella orientalis*	Hemiptera	Leaf	No data available	^27^
*Aspidiotus nerii*	Hemiptera	Leaf	No data available	^8^
*Planococcus harinoso*	Hemiptera	Leaf	No data available	^27^
*Pseudococcus spp*.	Hemiptera	Leaf	Sap exudation and piña discoloration leading	^23^
*Acutaspis agavis*	Hemiptera	Leaf	Leaf deformation, plant weakening, reduced photosynthesis, and possible plant death	^12^
*Caulotops agavis*	Hemiptera	Leaf	Plant weakening	^28^
*Dactylopius coccus*	Hemiptera	Leaf	No data available	^27^
*Rhopalosiphum maidi*	Hemiptera	Bud	No data available	^27^

* Observed infestations can reach up to 90% in Mexico, with reported damage of 24.5% in *A. tequilana* (Jalisco), 30% in *A. salmiana* (Hidalgo and Tlaxcala), and 40% in *A. fourcroydes* (Yucatán).

^†^ Observed damage was 79.9% in *A. potatorum* and 65.86% in *A. angustifolia*.

**Figure 1:**
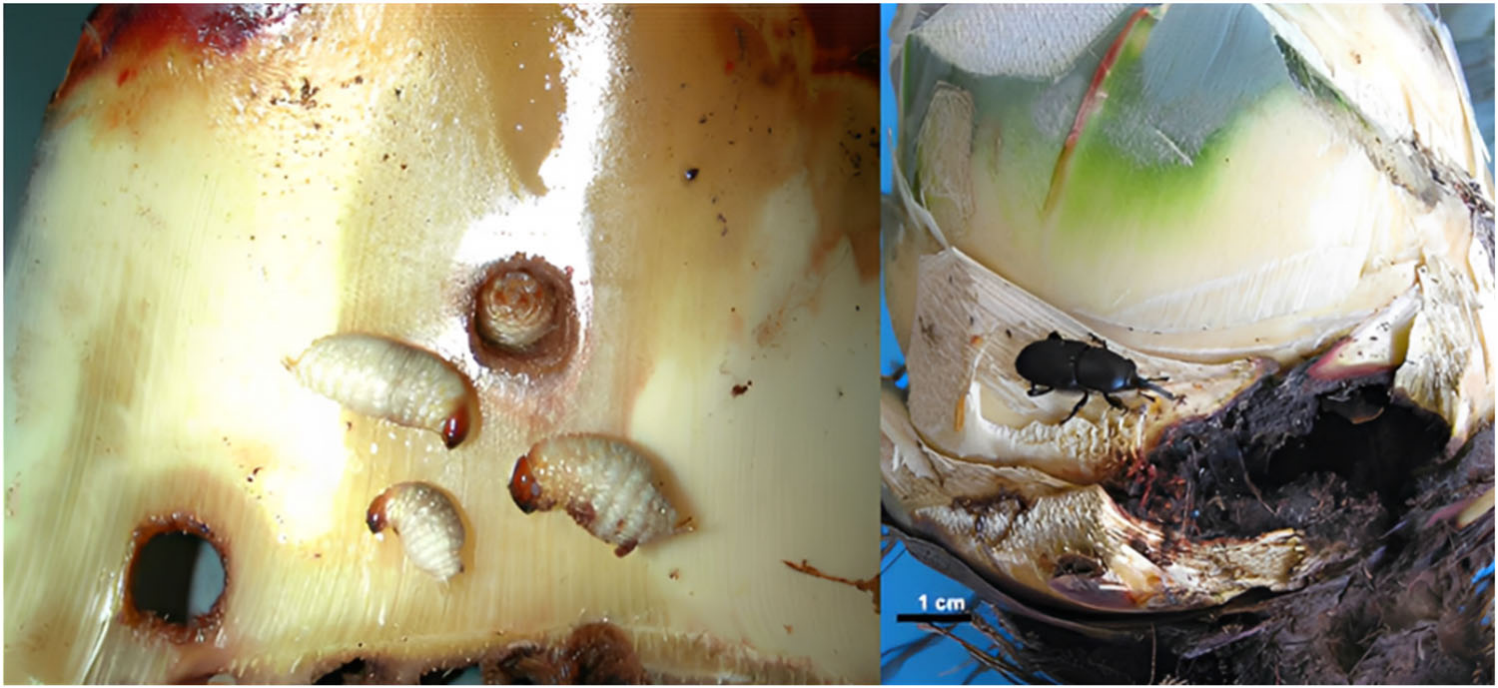
Damage to the agave piña (stem core) caused by agave weevil (*Scyphophorus acupunctatus*). Left: larval feeding damage characterized by internal tunneling and perforations in the stem core. Right: adult‐associated damage and external injury symptoms in agave tissues. Image reproduced from CESAVEG (2016).^32^

The main pest attacking agave crops is *Scyphophorus acupunctatus Gyllenhal* (Coleoptera: Dryophthoridae), also known as the agave weevil (Fig. 1).^10^, ^12^ It is native to Central and North America, although it also occurs in Europe, Asia, Africa, Oceania, and South America as a non‐native species.^29^ In Mexico, *S. acupunctatus* infestations can reach up to 90% in some regions,^9^ with reported damage include up to 24.5% *A. teqeuilana* plantations in Jalisco, 30% of *A. salmiana* in Hidalgo and Tlaxcala, and 40% of *A. fourcroydes* in Yucatán.^9^, ^30^, ^31^

Adult agave weevils typically inhabit the base of leaves and the interior of piñas (the central stem or core of the agave plant that stores large amounts of carbohydrates), where they form galleries of 25–40 individuals.^9^ Females oviposit in decomposing or water‐retaining tissues such as leaves and stems. After hatching, larvae excavate extensive galleries, severely compromising vascular integrity and structural stability.^13^, ^33^ Damage becomes evident through perforations and viscous secretions between the primary leaf meristem and leaves. Both adults and larvae reduce water and nutrient flow and cause mechanical injury by fracturing tissues, leading to plant weakening, lower piña quality, and often death.^9^, ^23^

Additionally, the weevil vectors phytopathogens such as *Pectobacterium carotovorum*, *Erwinia* spp., *Pseudomonas* spp., and *Aspergillus* spp., are associated with agave rot.^34^ Historically, control has relied on insecticides such as malathion and endosulfan. In semi‐arid regions, however, high temperatures, low humidity, and intense solar radiation reduce their effectiveness. The insect's concealed life cycle further limits pesticide contact, while biological agents, though effective in the laboratory, show limited success in the field.^26^, ^35^

**Figure 2:**
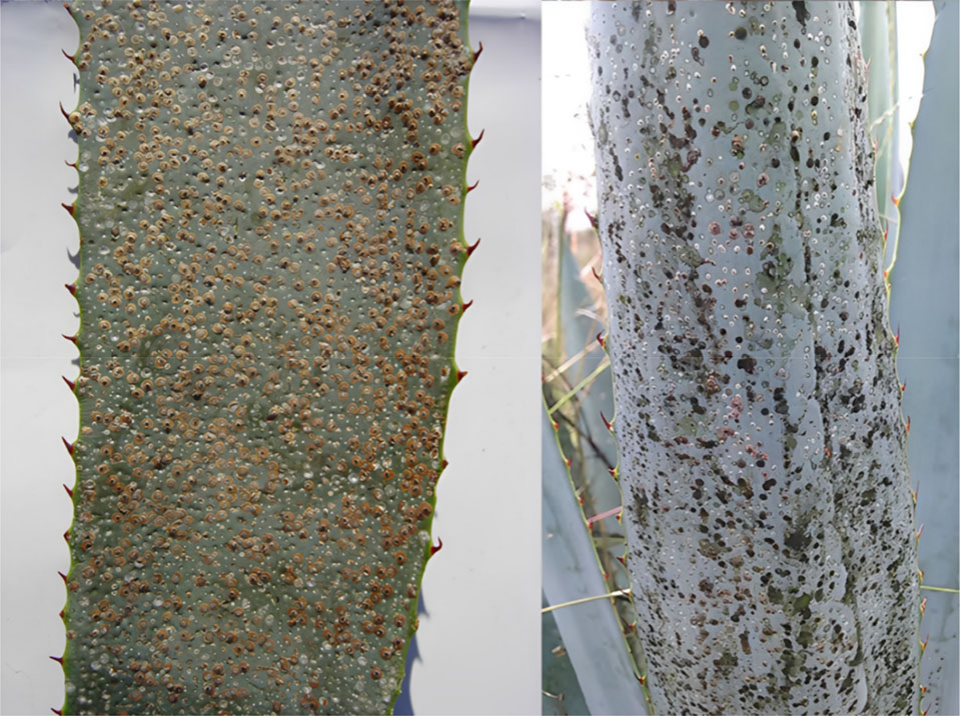
Infestation of *Acutaspis agavis* on agave leaves, showing scale insects attached to the leaf surface and associated tissue damage. Image reproduced from CESAVEG (2016).^32^

Another damaging pest is the armored scale *Acutaspis agavis* (Hemiptera: Diaspididae).^8^ Infestations can cover plants, block sunlight and reduce photosynthesis (Fig. 2). Symptoms include leaf deformation, plant weakening, and, in severe cases, death.^12^, ^33^ Protective scales, formed by waxes and exuviae, damage both leaf surfaces. Feeding on sap, the insect excretes honeydew that supports sooty mold (*Capnodium spp*.), further reducing photosynthesis.^23^ In tequila‐producing regions, control generally excludes insecticides, though severe infestations may require oils or contact chemicals.^12^

**Figure 3:**
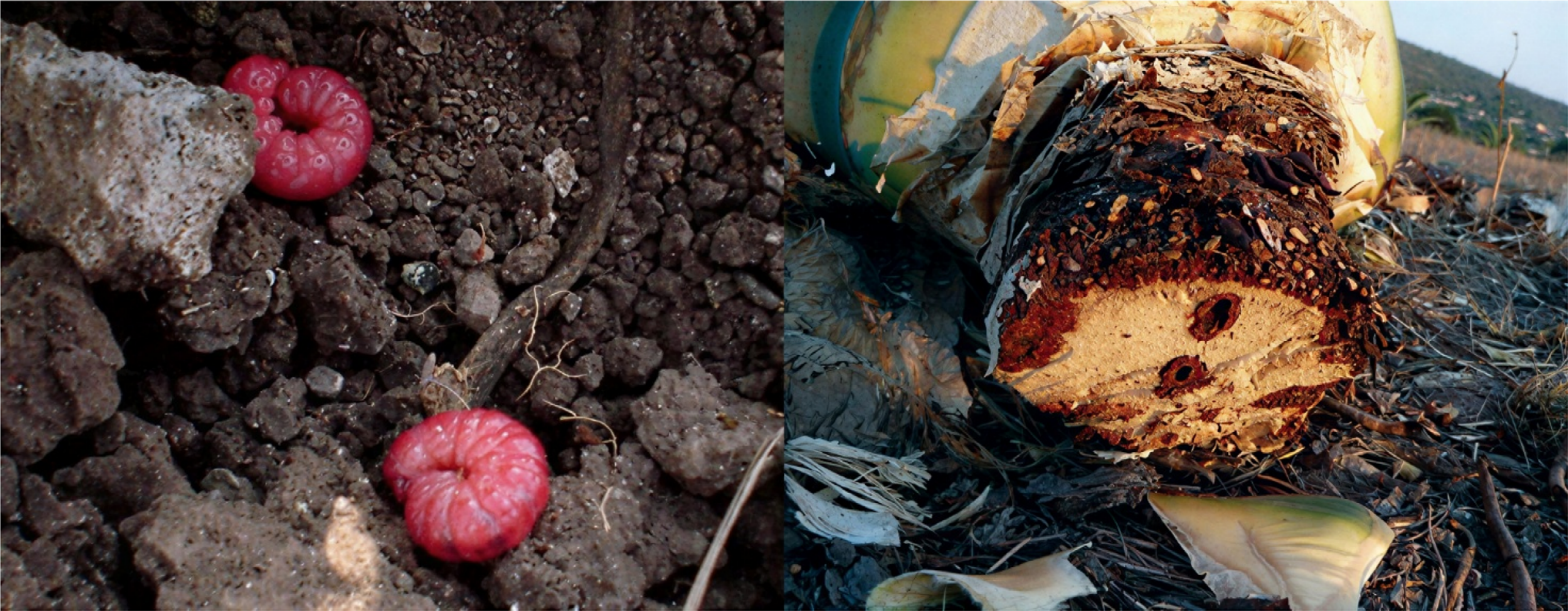
Larvae (left) and perforation damage (right) caused by *Comadia redtenbacheri* in the agave piña (stem core), illustrating tissue damage associated with larval feeding. Image reproduced from Queiroga (2020).^41^

*Comadia redtenbacheri* (Hammerschmidt, 1847) (Lepidoptera: Cossidae) (Fig. 3) is another pest insect that burrows into agave plants during its larval stage. Its life cycle lasts approximately 1 year, but adults have a short lifespan, living only between 3 and 5 days due to their underdeveloped mouthparts, which prevent them from feeding.^36^, ^37^ During agave colonization, these insects form colonies of 40–60 larvae in the center of the stem, near the junction with the fleshy leaves.^11^ After hatching, the larvae migrate to the rhizome and go through seven instars in about 8 months.^37^ The galleries created by the larvae result in the reduction of the root tissue, leading to signs of weakness such as yellowed or dried leaves, as well as a decrease in the height and vigor of the agaves. In severe cases, this infestation can even lead to the death of the plant.^38^

The management of *Comadia redtenbacheri* presents significant challenges due to their endophytic development, which protects them from external interventions. This trait, combined with their high reproductive rate and rapid dispersal, compromises the effectiveness of chemical treatments and promotes the persistence of infestations in the field.^11^, ^39^ Furthermore, in some regions of Mexico, the traditional use of these insects as a food resource adds additional complexities to control strategies.^40^

## 3 CRY TOXINS: MECHANISM AND SPECIFICITY

Given the economic impact of agave pests and the limitations of current management strategies, alternative approaches for pest control are required. Transgenic plants producing *Bacillus thuringiensis* Cry proteins are among the most effective and environmentally safe strategies for pest control.^42^ *B. thuringiensis* is a gram‐positive, aerobic bacterium commonly found in soil, insects, grains, and aquatic environments.^43^ Identified in 1901 and rediscovered in 1911 for its insecticidal properties, *Bt* gained commercial relevance in 1938.^44^, ^45^ The bacterium produces Cry proteins, insecticidal toxins with a conserved three‐domain structure. Classified into families such as Cry1 and Cry2, these proteins are essential in integrated pest management.^46^, ^47^ According to official nomenclature, Cry toxins are parasporal inclusion proteins from *Bt* with confirmed pesticidal activity or sequence similarity to characterized toxins.^48^

**Figure 4:**
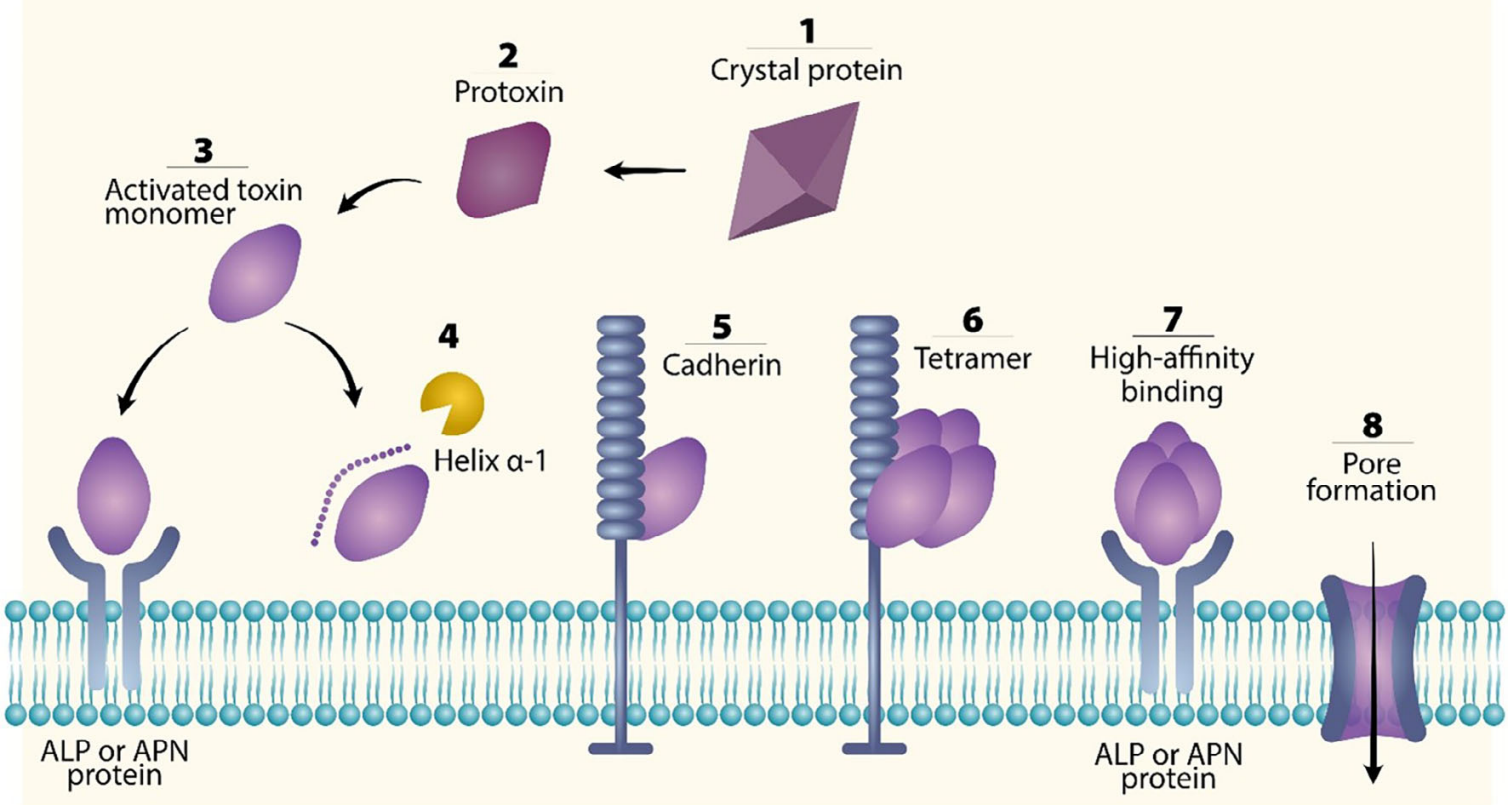
Mode of action and specificity of Cry toxins produced by *Bacillus thuringiensis*. (1) Cry toxin crystals are ingested by susceptible insects and solubilized in the alkaline environment of the midgut. (2) Solubilized protoxins are activated by intestinal proteases through cleavage of N‐ and C‐terminal regions. (3) The activated toxin monomer binds with low affinity to aminopeptidase N (APN) or alkaline phosphatase (ALP) receptors on the epithelial cell membrane. (4) Subsequent cleavage of helix α‐1 enables (5) binding to cadherin‐like receptors, promoting (6) oligomerization of the toxin. (7) The oligomeric toxin binds with high affinity to APN or ALP receptors. (8) Finally, the oligomer inserts into the membrane, forming pores that disrupt cellular homeostasis and lead to cell lysis. This mechanism is particularly relevant for lepidopteran and coleopteran larvae associated with agave crops, which possess an alkaline midgut environment that facilitates Cry toxin activation.

The mode of action of Cry proteins (Fig. 4) involves receptor‐mediated toxicity in the insect midgut.^47^, ^49^ After ingestion, Cry protoxins are solubilized under alkaline conditions and activated by proteases.^50^ The resulting toxins bind to receptors such as aminopeptidases (APN), alkaline phosphatases (ALP), cadherins, and ABC transporters, triggering oligomerization, membrane insertion, and pore formation, ultimately leading to epithelial cell lysis.^51^, ^52^, ^53^, ^54^ Midgut pH and receptor availability are central determinants of Cry protein activity, with alkaline conditions facilitating protoxin solubilization and activation,^55^ while the composition and distribution of epithelial receptors govern binding dynamics and toxin specificity.^56^

Given that coleopteran insects constitute the primary pests affecting agave crops, Cry3 proteins are of particular relevance. Members of this group, including Cry3A, Cry3B, Cry3Bb, and Cry3Ca, exhibit specificity toward beetles^57^, ^58^ and have been successfully deployed in transgenic crops. Field evaluations of *Bt* maize expressing Cry3Bb1 (e.g., event MON863) have demonstrated high efficacy against *Diabrotica virgifera virgifera*, with larval mortalities approaching 98.5% and significant reductions in adult emergence across multiple locations and years.^59^ These outcomes highlight the robustness of Cry3‐mediated control under agronomic conditions. Notably, bioassays of transgenic cotton plants co‐expressing *Cry3* and *Cry3Bb1*, genes primarily active against coleopteran pests, demonstrated substantial insecticidal activity against lepidopteran larvae, with *Pectinophora gossypiella* and *Helicoverpa armigera* exhibiting mortalities of approximately 60% and 75%, respectively, illustrating the broader spectrum achievable through combinatorial Cry expression.^60^

Consistent with field and bioassay observations, the potency of Cry3 proteins against coleopteran pests is underpinned by larval midgut physiology, wherein neutral to alkaline conditions facilitate protoxin solubilization and activation.^61^, ^62^ This physiological compatibility underlies Cry3 activity across diverse coleopteran families, including Chrysomelidae, Scarabaeidae, Tenebrionidae, and Curculionidae,^63^ where susceptibility is further corroborated by molecular, physiological, and experimental evidence. Within Curculionidae, Cry3Aa‐binding proteins, such as aminopeptidase N, have been identified in larval midgut tissues of *Rhynchophorus ferrugineus*.^64^ In addition, bioassays using bacterial isolates have demonstrated high susceptibility of this species to *Bacillus thuringiensis*, with mortality rates reaching 100% at 1 × 10^8^ CFU mL^−1^ within 4 days post‐treatment, highlighting the strong insecticidal potential of *Bt* against curculionid pests.^65^

Similarly, in *Premnotrypes vorax*, an important coleopteran pest of potato crops, both native and recombinant Cry3Aa proteins have shown direct toxicity in feeding assays, resulting in larval mortality rates of approximately 52–57% after 5 days of exposure.^66^ Transcriptomic analyses of this species have also revealed homologs of key Cry receptors, such as cadherins, APN, ALP, and ABC transporters, supporting the mechanistic basis for Cry susceptibility.^67^ Collectively, these findings substantiate the activity of Cry proteins against coleopteran pests, including members of Curculionidae. Given that *Scyphophorus acupunctatus* belongs to the same family, these observations suggest that Cry proteins may be effective against this species, although its susceptibility remains to be experimentally validated.

Lepidopteran insects are well‐established targets of *Bacillus thuringiensis toxins*, with Cry1 and Cry2 proteins exhibiting high toxicity across multiple families, including Noctuidae, Tortricidae, Plutellidae, Chrysomelidae, and Geometridae.^68^, ^69^ Despite the extensive characterization of Cry activity within Lepidoptera, susceptibility data for Cossidae remain scarce, and no experimental evidence is currently available for *Comadia redtenbacheri*. In contrast, the efficacy of Cry proteins against Hemiptera is markedly reduced, a pattern attributed to gut physiological constraints, including neutral to acidic conditions and distinct proteolytic enzyme profiles that compromise toxin solubilization and activation.^70^, ^71^ A similar lack of evidence is observed for *Acutaspis agavis*, for which Cry susceptibility has not yet been experimentally assessed.

This differential susceptibility across insect orders underscores the intrinsic selectivity of Cry proteins, which constitutes a key advantage for their application in crop protection. In *Agave* spp., this selectivity is particularly relevant given the predominance of coleopteran pests and the ecological sensitivity of semi‐arid production systems, where minimizing impacts on non‐target organisms is essential. By selectively targeting key pests such as the agave weevil, Cry proteins represent a biologically precise and environmentally compatible strategy for pest management under these conditions.

From a biotechnological perspective, *Cry* genes have been extensively deployed in commercial transgenic crops. Genes such as *Cry1Ab*, *Cry1F*, *Cry1Ac*, *Cry2Ab2*, *Cry1Ab‐Ac*, *Cry2Ae*, *Cry1Fa2*, *Cry1A.105*, *Cry1B.868*, and *Cry1Da_7* are used for resistance to lepidopteran pests, while *Cry3A*, *Cry3Bb1*, *mCry3A*, and *Cry34Ab1‐Cry35Ab1* target coleopterans, and *mCry51Aa2* targets hemipterans. However, most commercial transgenic crops with *Cry* genes primarily target lepidopterans, whereas only a limited number of genetically engineered crop events targeting other insect orders have been commercially approved.^72^ A notable example of this technology is the GM soybean Intacta RR2 PRO® (MON 87701 × MON 89788) that expresses the *Cry1Ac* gene and is glyphosate‐tolerant, revolutionizing pest management in South America by protecting over 30 million hectares from defoliators like *Anticarsia gemmatalis*, *Chrysodeixis includens*, *Chloridea virescens*, and *Helicoverpa armigera*.^73^, ^74^

However, the widespread adoption of Cry‐based technologies has been accompanied by the emergence of resistance in several pest species, compromising long‐term efficacy. For example, *Spodoptera frugiperda* developed resistance to *Cry1F* in Puerto Rico by 2010, and *Helicoverpa armigera* resistance in China rose to 4.7% by 2017.^75^, ^76^ To minimize this, resistance management strategies such as planting refuge areas of non‐GM crops are recommended to maintain susceptible pest populations. However, growers often reduce the size of these refuge areas in pursuit of short‐term gains, which can accelerate the evolution of resistant insect populations. Furthermore, maintaining high toxin concentrations in plant tissues is essential to ensure the high‐dose requirement, which aims to eliminate more than 95% of resistant individuals, as recommended by the US Environmental Protection Agency.^77^ To this end, researchers are improving Cry potency through gene stacking, domain swapping, targeted amino acid substitutions, and proteolytic activation site incorporation.^78^, ^79^, ^80^, ^81^, ^82^

**Gene pyramiding** or **stacking** is a key approach to managing resistance to *Bt* toxins. It involves combining two or more distinct genes targeting the same pest, ideally without cross‐resistance, to achieve ‘redundant killing.’ This ensures insects that are resistant to one toxin are controlled by another, leaving only individuals homozygous to resistance to all toxins with a high chance of survival.^78^ This method reduces the prevalence of resistant populations, particularly in areas not previously exposed. Successful applications include Bollgard II (*Cry1Ac* + *Cry2Ab*) and Bollgard III, which added *Vip3A* for broader protection.^83^ However, even pyramid events like MON89034 (*Cry1A.105* + *Cry2Ab2*) may eventually encounter resistance, highlighting the complexity of sustainable pest management.^84^

**Targeting *Cry* genes to chloroplasts** enhances protein accumulation and biosafety. Chloroplast transformation allows high‐level, stable expression, with up to 10 000 copies per cell, while maternal inheritance reduces transgene spread.^85^ Chloroplast targeting can be achieved directly via homologous recombination or through nuclear transformation with signal peptides.^86^ Studies in rice and cotton have shown that chloroplast‐targeted Cry proteins can increase expression dramatically, improving resistance to lepidopteran pests while addressing biosafety concerns.^81^, ^87^

**Proteolytic activation** of Cry protoxins by insect gut proteases is essential for toxicity. Variations in protease activity can influence resistance. Engineering Cry toxins with optimized cleavage sites, such as *mCry3A* with a chymotrypsin/cathepsin G site, enhances processing and activity, providing more effective control against pests like *Diabrotica virgifera virgifera*. This variant has been widely deployed in commercial maize events.^88^

**Directed site‐specific mutagenesis** allows precise modification of amino acids in Cry toxins, particularly in domain II, which mediates receptor binding. Such modifications can increase or reduce toxicity depending on the target pest.^89^ Recent studies on *Cry1Ac* and *Cry2Aa* demonstrate that strategic amino acid changes can enhance mortality rates against pests like *Spodoptera litura*, emphasizing the potential of mutagenesis for pest‐specific optimization.^80^

**Chimeric Cry proteins** combine domains from different *Bt* toxins to generate new insecticidal specificities. Domain swaps, especially in domain III, have produced proteins with higher toxicity than parental forms.^90^ Examples include *Cry1A.105* (used in MON89034) and *eCry3.1Ab*, which targets coleopteran pests like the western corn rootworm. Chimeric strategies expand the spectrum of pest control and could be adapted to crops such as *Agave*, potentially extending protection to other insect orders, including hemipterans.^91^, ^92^

## 4 CHALLENGES AND FUTURE DIRECTIONS FOR CRY TECHNOLOGY IN *AGAVE*

Although Cry proteins have been extensively studied in crops, genetic transformation of monocotyledons is often hindered by species‐intrinsic traits, such as limited *in vitro* regeneration capacity, fewer competent cells in the target tissue, reduced production of phenolic compounds that activate virulence (*vir*) genes, and physical cell‐wall barriers.^93^ These constraints are reflected in the low and highly variable transformation efficiencies reported across monocot species (Table 2). Moreover, *Agrobacterium*‐mediated transformation in monocots is restricted to a narrow range of genotypes, and the effectiveness of the technique is further limited by the recalcitrance of many genotypes.^94^

**Table 2:** Overview of genetic transformation efficiencies in monocot species: methodological approaches, explant sources, and reported outcomes

Species	Method	Explant	Efficiency (%)	Reference
*Oryza sativa*	*Agrobacterium*	Callus	Up to 46	^95^
*Zea mays*	*Agrobacterium*	Immature embryos	5.5	^96^
*Triticum aestivum*	*Agrobacterium*	Immature embryos	5–25	^97^
*Hordeum vulgare*	*Agrobacterium*	Immature embryos	4–36	^98^
*Sorghum bicolor*	*Agrobacterium*	Immature embryos	0.8–3.5	^99^
*Sorghum bicolor*	*Agrobacterium* with morphogenic genes	Immature embryos	Up to 30.8	^100^
*Saccharum* spp.	*Agrobacterium*	Embryogenic callus	2.2	^101^
*Panicum virgatum*	*Agrobacterium **with** morphogenic genes*	Immature leaf segments	3–6	^102^
*Miscanthus sinensis*	*Agrobacterium*	Embryogenic callus	1.05	^103^
*Eleusine coracana*	Agrobacterium	Shoot apical meristems	0.6–11.8	^104^
*Avena sativa*	*Agrobacterium*	Immature embryos	1.1–12.3	^105^
*Triticum monococcum*	Biolistic	Immature embryos	0.3–0.8	^106^
*Agave salmiana*	*Agrobacterium*	Embryogenic callus	2.7	^107^
*Agave sisalana* ‘RLV 19’	*Agrobacterium*	Meristems	0.5	^108^

In plants, genetic transformation is primarily achieved using *Agrobacterium*‐mediated or biolistic methods. *Agrobacterium* typically enables more precise, low‐copy insertions, but its efficiency can be limited in recalcitrant species. In contrast, biolistic approaches bypass host–pathogen compatibility barriers, although they are often associated with multiple insertions and genomic rearrangements.^109^ More recently, alternative delivery systems, such as morphogenic regulator‐assisted transformation (e.g., *Wuschel* and *Baby boom*), have shown potential to improve transformation efficiency and regeneration capacity in some monocots. These regulators promote cell dedifferentiation and enhance regeneration competence, thereby facilitating the recovery of transformed plants.^110^

For instance, Fontanet‐Manzaneque *et al*. demonstrated that a system incorporating *Wus*/*Bbm* achieved a transformation efficiency of 30.8% in sorghum, a highly recalcitrant species, reinforcing the potential of this approach to overcome key barriers in difficult‐to‐transform crops. However, such improvements are not universal.^100^ In switchgrass (*Panicum virgatum*), a highly polyploid species, transformation efficiencies of approximately 3–6% have been reported depending on ploidy level and genotype, even when morphogenic regulators are used to enhance regeneration competence.^102^ These contrasting outcomes highlight that, while morphogenic regulators can significantly improve regeneration capacity, their effectiveness remains strongly dependent on species‐specific factors.

Emerging genome editing technologies provide additional tools for genetic improvement. CRISPR/Cas‐based systems enable precise and targeted modifications and may reduce reliance on stable transgene integration, particularly when combined with transient expression or DNA‐free delivery approaches.^111^ Nevertheless, their application still depends on effective delivery and regeneration systems, which are key determinants of success across monocot species.

In *Agave* spp., efforts to establish transformation systems have achieved limited success, generally with reduced efficiencies. A pioneering patent describes the transformation of *Agave tequilana ‘Weber Azul’* using the biolistic technique with plasmid DNA containing marker genes (*Bar* and *hpt* – selectable genes/*uidA* – reporter gene). However, the patent does not report transformation efficiency.^112^ Similarly, Flores‐Benítez *et al*. outlined a protocol for somatic embryogenesis from undifferentiated callus derived from *Agave salmiana*. The authors used cocultivation with *Agrobacterium tumefaciens* carrying the reporter gene *uidA*. This approach resulted in the regeneration of four transformed explants via organogenesis, corresponding to a transformation efficiency of 2.7%.^107^

In another study, Aguilar *et al*. reported the *Agrobacterium tumefaciens*‐mediated transformation of meristem explants from *Agave tequilana* and *Agave desmettiana*, followed by plant regeneration via organogenesis. Although the authors described the methodology in detail, no transformation efficiency was reported, limiting the quantitative assessment of the protocol's success.^113^ In the same way, Gao *et al*. developed a transformation system for *Agave* (hybrid 11 648) using cocultivation of callus with *A. tumefaciens* to express hevein‐like peptides for increased resistance to *Phytophthora nicotianae*. From 150 explants, 37 lines were obtained, with seven confirmed by PCR and Southern blot.^114^ These studies demonstrate that genetic transformation of agave is feasible, however progress remains limited due to the small number of studies and the scarcity of quantitative data, such as transformation efficiency and regeneration rates.

Despite the potential for genetic transformation, interest in developing commercial transgenic agave remains limited, with significantly lower investment compared to other semi‐arid crops. In particular, no studies have yet investigated the application of Cry proteins in *Agave* spp. Nevertheless, the successful use of Cry technology in non‐conventional crops cultivated in semi‐arid regions underscores its broader applicability. For instance, the *Cry2Aa* gene was successfully introduced into *Miscanthus lutarioriparius*, a perennial rhizomatous grass species from China, considered a promising bioenergy crop for biomass production. The expression of the *Cry2Aa* gene in this species demonstrated effective insecticidal activity, providing a sustainable pest control alternative in bioenergy systems adapted to marginal lands.^103^ Similarly, the *Cry1Ab* gene was introduced into pigeon pea (*Cajanus cajan*), a legume widely cultivated in tropical semi‐arid regions and valued for its high‐quality protein. Transgenic lines expressing *Cry1Ab* exhibited significant toxicity against the lepidopteran pest *Helicoverpa armigera*, with 90% larval mortality rate *in vitro* bioassays,^115^ highlighting the capacity of *Bt* technology to enhance pest resistance in underutilized legume crops cultivated under challenging environmental conditions. Collectively, these findings indicate that *Bt* genes can confer effective and robust pest control even in marginal, semi‐arid systems, reinforcing the feasibility of translating similar strategies to agave production.

Concurrently, developing GM agave must navigate regulatory, phytosanitary, and socioeconomic constraints that could influence adoption. In Mexico, GM organisms are regulated under the Biosafety Law of Genetically Modified Organisms (LBOGM), requiring environmental and biosafety assessments and approval by national authorities.^18^ Agave‐derived products are also subject to denomination of origin protections, for example tequila production is governed under NOM‐006‐SCFI, which defines the geographic production areas across specific municipalities in the states of Jalisco, Guanajuato, Michoacán, Nayarit, and Tamaulipas, and establishes certification requirements for *Agave tequilana* Weber Blue.^116^, ^117^ Similar considerations apply to export‐oriented industries such as Brazilian sisal, where international regulations and consumer acceptance may affect adoption.

Lessons from other transgenic crops in Mexico illustrate key challenges for adoption. For example, transgenic cotton, authorized in 2010, benefited from its predominantly self‐pollinating nature, which minimizes gene flow. In contrast, transgenic soybean, authorized in 2012, faced revocation of its license in 2017 due to unauthorized cultivation and pollen contamination of honey.^18^ These experiences highlight the importance of considering both biological traits and socio‐economic factors in deployment strategies. In the case of agave, commercial fields are propagated mainly through vegetative offshoots, and flowering is often suppressed, resulting in negligible natural pollination and potentially limited gene flow from modified plants.^118^ Nevertheless, these biological advantages do not replace the need for thorough regulatory evaluation, engagement with local communities, and careful market assessment when planning the introduction of GM agave.

For smallholder farmers, who make up the majority of agave producers in many regions, the decision to adopt GM agave will depend not only on regulatory and market conditions but also on economic feasibility, accessibility, and clear agronomic benefits. At the same time, implementation of Cry‐based technologies must account for evolving pest pressures, as illustrated by the agave weevil, which has recently been reported beyond its traditional range, including parts of Europe, highlighting emerging phytosanitary challenges.^29^ Therefore, deploying *Bt*‐based interventions in *Agave* spp. requires a multidimensional approach that integrates environmental risk assessment, compliance with domestic and international regulations, pest management effectiveness, and socioeconomic considerations, ensuring that these technologies are both effective and sustainable within the constraints of semi‐arid agricultural systems.

## 5 PERSPECTIVE ON *BT* TECHNOLOGY IN AGAVE PLANTS

The rapid expansion of semi‐arid regions worldwide, intensified by climate change, is increasingly constraining agricultural productivity and resilience, leaving little room for incremental solutions and demanding coordinated, transformative strategies. In this scenario, *Agave* spp. stands out as one of the most promising crops for the future of agriculture under extreme environmental conditions. Its exceptional water‐use efficiency and inherent tolerance to climatic stress position *Agave* species not only as a traditional source of high‐value fibers and beverages, but also as a strategically relevant feedstock for bioenergy and bio‐based value chains. Few perennial crops combine such physiological resilience with the capacity to contribute simultaneously to food, fiber, and energy systems, supporting diversified and sustainable agricultural transition.

Yet, the full potential of agave cultivation is fundamentally constrained by vulnerability to insect pests, which directly undermine productivity, economic viability, and the long‐term sustainability of these systems. As discussed above, insect pressure directly undermines the productivity, economic viability, and long‐term sustainability of agave‐based systems. Addressing insect damage is therefore a critical prerequisite for realizing the full agronomic and economic value of *Agave* spp., particularly in semi‐arid regions where alternative cropping options are limited.

Our research group has focused on the development of transgenic agave plants with glyphosate resistance,^108^ alongside broader efforts to enhance tolerance to both biotic and abiotic stresses. From this work, several key lessons have emerged. First, transformation outcomes are highly genotype‐dependent, with some cultivars proving recalcitrant to standard protocols. Second, regeneration efficiency remains a major bottleneck, compounded by the limited availability of high‐quality explant material. Third, high endophytic microbial loads and tissue oxidation further complicate tissue culture and transformation efforts. Together, these challenges highlight the technical complexity of genetic improvement in *Agave* spp. and underscore the need for optimized, species‐specific protocols to achieve stable and reproducible transgenic events. These practical challenges underscore why, despite proof of concept, genetic transformation protocols remain insufficiently optimized for commercially important species.

Genetic transformation protocols remain insufficiently optimized for commercially important species such as *A. tequilana*, central to beverage production, and *A. sisalana*, the primary source of natural hard fibers. Beyond technical constraints, regulatory, socio‐cultural, and market‐related considerations, particularly in regions where *Agave* spp. is closely linked to cultural heritage and geographical indications, represent additional challenges that require transparent, science‐based engagement. Continued underinvestment in agave biotechnology effectively perpetuates a production model that is vulnerable to pest outbreaks, increasingly costly to manage, and progressively misaligned with environmental and economic sustainability goals. From this perspective, the development of *Bt* agave emerges as a compelling and largely unexplored opportunity to strengthen pest management and agricultural resilience in semi‐arid systems where few alternative crops can thrive.

Nonetheless, semi‐arid cultivation presents particular challenges for pest management, including water limitation, simplified cropping systems, and climatic variability, which can complicate the deployment of integrated pest management strategies.^119^ Evidence from other dryland crops demonstrates that transgenic *Bt* technologies can be effectively implemented under such conditions. For example, *Bt* cotton in semi‐arid regions of Pakistan maintained high productivity and achieved a benefit–cost ratio of 1.55. In general, *Bt* genotypes showed higher yields than non‐*Bt* counterparts, with soils cultivated with *Bt* plants exhibiting greater availability of N, P, and Zn.^120^ The predominance of monoculture in agave cultivation similarly complicates resistance management, underscoring the importance of context‐sensitive, ecologically informed strategies to ensure the long‐term efficacy of Bt‐based pest control in semi‐arid production systems.

This opportunity gains further relevance under scenarios of escalating climate risk and rising global demand for biomass to support low‐carbon energy transitions. We thus call for a coordinated and forward‐looking effort to advance agave research, accelerating knowledge generation, rapidly developing genetic, molecular, and functional knowledge regarding the plant, creating robust and efficient transformation systems to enable genetic improvement, and assessing the use of *Bt* proteins within integrated pest management frameworks to handle pests sustainably. This is not merely an exercise in technological innovation, it is a strategic opportunity to reposition agave as a modern, climate‐resilient crop aligned with the demands of sustainable agriculture and bioenergy systems.

## AUTHOR CONTRIBUTIONS

This work was funded by Shell Brazil and the National Agency of Petroleum, Natural Gas and Biofuels. The authors declare no relevant financial or non‐financial interests. All authors contributed to the conception and design of the study. Aline Vitória Corim Marim was responsible for material preparation, data collection, and analysis, as well as drafting the first version of the manuscript. All authors reviewed previous versions and read and approved the final manuscript.

## Data Availability

The initial datasets generated and analyzed for this perspective article are not publicly available due to their preliminary nature, but they can be obtained from the corresponding author on reasonable request.
